# Community Sport and the Politics of Aging: Co-design and Partnership Approaches to Understanding the Embodied Experiences of Low-Income Older People

**DOI:** 10.3389/fsoc.2019.00005

**Published:** 2019-02-12

**Authors:** Louise Mansfield, Tess Kay, Nana Anokye, Julia Fox-Rushby

**Affiliations:** ^1^Department of Life Sciences, Brunel University London, Uxbridge, United Kingdom; ^2^Department of Primary Care and Public Health Sciences, King's College London, London, United Kingdom

**Keywords:** community sport, aging, coproduction, embodiment, physical activity

## Abstract

The promotion of physical activity for older people is dominated by biomedically informed polices emphasizing the prescription of exercise as medicine and a universal approach to the promotion of active aging in later life. Yet, more recent research recognizes that being physically active in later life is complex and contested, shaped by the intersections of biological, psychological, and sociological experiences, and requires differentiated responses that address this complexity. There is a disconnect between research, policy, and the physical activity experiences of older people which leads to over-generalized policy and practice in the promotion and delivery of community sport to older people. This paper presents findings from a complex community sport project employing a coproduction framework with low income older age people. Participatory community approaches including focus group discussions, and extended observations and informal conversations throughout the project develop understanding of the complexities of aging and community sport engagement among older people with limited income. Three themes are identified and discussed: (1) lived experience, aging bodies, and the changing dynamics of involvement in sport and exercise in the life course, (2) embodying aging—moving beyond practical barriers for understanding aging, lived experience and being physically active, and (3) corporeal pleasures of older sporting bodies. The paper concludes that there is a need to explore the significance of locally specific public knowledge from older people which directly addresses the complexity and inequalities of individuals' everyday lives in their communities; lived experiences likely to impact on preferences for, engagement in, and enjoyment of physical activity.

## Introduction

The global population is aging with implications for all sectors in society including public health, physical activity, and community sport. According to the United Nations (UN) worldwide, the numbers of older people (aged 60 years and above) is likely to rise from 901 million to 1.4 billion between 2015 and 2030 and by 2050 the global population of older people could more than double to 2.1 billion. Moreover, numbers of those categorized as the oldest old (80 years and over) will have tripled between 2015 and 2050 to 434 million (United Nations, 2015). A deficit social and political model of aging, driven by concerns about older peoples' withdrawal from social interaction and activity and their growing dependence on the State has shaped the politics of aging in the UK and other socio-economically similar counties. An exaggerated pessimism about aging has prevailed. The assumption has been that increasing numbers of older people will put undue pressures on public expenditure particularly in terms of health and social welfare (Cardona, 2008; Asquith, 2009). A wider ethic of individual responsibility for health has suffused UK Government policy on aging. There has been, over the past two decades, a predominant policy narrative based on ideas of successful aging which emphasize that acceptable older age citizenship requires a self-directed lifestyle ensuring independence, mobility, health, quality of life, and consumption (Katz, 2001; Cardona, 2008). As Katz (2001, p. 28) argues, “timelessness” has become a political, social, and economic tool for promoting every aspect of life including leisure, education, and health promotion as a “*lifelong* project.”

It is unsurprising that physical activity has been uncritically conflated with healthy aging in both social and political thought. Physical activity policy, promotion and practice is underpinned by a well-established scientific evidence base for the physical and mental health benefits of being active and an equally authoritative science endorsing physical activity in the prevention of chronic disease (World Health Organization, 2010; Department of Health, D. H., 2011). It is also significant that the promotion and prescription of physical activity alongside the surveillance and monitoring of health has a long history in the development of the modern public health movement and the associated political debates. Against a backdrop of Nineteenth century concerns about sanitation and personal hygiene coupled with a later prioritization of national fitness in several countries worldwide, physical activity has developed as a principal cultural context for population level health education and lifestyle improvement (see for example Riordon, 1980; Lupton, 1995; Howell and Ingham, 2001; McPhail, 2009; Shilling, 2012). Set in the contemporary context of an aging population and evidence of a decline in physical activity in old age, physical activity is being promoted as a panacea for the problems of old age including illness, frailty, disability and loneliness, and a universal means of achieving a healthy, disability free life into old age. UK policy recommendations for older people represent a one size-fits all approach which is largely inappropriate for the needs of many people in old age (Kay, 2016). There is a prevailing politics of active aging serving to reinforce sport and exercise as the solution to the health and well-being problems of old age (Stenner et al., 2010; Pike, 2011). Active living is not simply presented as the preserve of the young; it is a lifelong project and now hailed as the key to successful and healthy aging. However, both aging and the role of physical activity in successful aging are contested. Whilst physical, psychological and social limitations in old age can be restrictive, problems in old age are not inevitable or ubiquitous (Bowling, 2005). Physical activity is not a panacea for the treatment and prevention of all known diseases, nor can engagement in physical activity be guaranteed to preserve youth, health, and vitality in an aging population. Indeed, physical activities including sporting endeavors may well be performed and enjoyed by many older people (Phoenix and Tulle, 2017) but they are not necessarily accessible, acceptable, or appropriate for the aging population as a whole. Furthermore, engaging in sport in later life reflects complex negotiations of the aging process including simultaneous experiences of resistance, redefinition, and acceptance (Dionigi et al., 2013).

As more people live longer, it is understandable that questions are raised about whether and how people can live a high-quality life in their older years. There are many gaps in our knowledge about the contribution and costs of aging populations, patterns and experiences of quality of life, and effective interventions for healthy aging be they clinical, pharmacological, or community-based (Beard and Bloom, 2015). Chronological aging leads to biological decline, but the nature, extent, and impact of advancing years differs amongst individuals and communities. Functional decline varies between people in old age and the balance of influence between genetic and environmental factors continues to be debated (Christensen et al., 2000). Health behaviors and social inequalities also contribute to the quality of life in later years (Suzman et al., 2015). The social gradient of health and illness is well-known. Living with socioeconomic disadvantage is associated with worse health across the life course (Marmot, 2005) and it is the case that social conditions influence older peoples' abilities to access and engage in physical activity (Kay, 2016). Yet little understanding from social science perspectives is embedded into policies about older people (Bambra, 2011). Missing from policy on aging and physical activity is recognition of the lived experience of aging and the significance of embodiment in understanding aging; a perspective identified as aging embodiment which frames the approach in this study (Tulle and Krekula, 2013). The established and diverse literature and theoretical insights about embodiment indicate it as a concept for understanding the ways that human bodies give form to socio-cultural and political beliefs and issues such as gender, disability, race and ethnicity, social class and age, and the intersections of such ideals (Featherstone et al., 1991; Williams and Bendelow, 1998; Turner, 2008). Central to theories of embodiment are issues of corporeal power focusing on the body as a basis for social order, control and constraint, and opportunity, resistance, and negotiation (Shilling, 1991). Reflecting the wider scholarship on embodiment, work on aging embodiment has shifted away from an established focus on biology as a reference point for aging. Rather, there is recognition of the significance of social aspects of corporeality, particularly in relation to experiences of aging, the positioning of aging bodies and the negotiation of meaning and self-identity over time (Gilleard and Higgs, 2014). The body is fundamental to the way older people participate in social life and in sport and physical activity and a sense of self in old age develops in connection to the relationships experienced in social contexts. Given the entrenched rhetoric of decline in older age, and the lack of policy recognition about the complexities of aging, it is tempting to view aging identities developed through sport and physical activity simply through the lens of control. However, processes of adjustment, integration, and negotiated responses characterize embodied identities across the lifecourse and in different social contexts (Featherstone and Hepworth, 1998; Shilling, 2008; Tulle and Krekula, 2013; Phoenix and Tulle, 2017). It is with such nuanced thinking about the interplay between aging bodies and corporeal negotiation, suggested in the aging embodiment literature, that the discussion in this paper is framed. At the community level, thinking with a sensitivity to complex relations of power, aging and the body allowed us to examine peoples' capacity to influence organizations and institutions which impact on their lives and specifically in relation to peoples' ability to determine the character of community sport activities and the nature of their own engagement in them (Tomlinson, 1998).

In responding to the criticisms of the politics of aging and active aging we have introduced, this paper discusses the qualitative findings of a complex community sport project delivered with low income older age people (60+ years) living in the London (UK) Borough of Hounslow between 2013 and 2017. The study was part of a wider community sport project; The Health and Sport Engagement (HASE) project which co-designed, delivered and evaluated the physical activity, health and well-being impacts of community sport with a range of groups who self-identified as inactive (see Mansfield et al., 2015). In this paper, we explore the findings from our work with low income older age people living in sheltered accommodation to co-design a community sport project for people in later life. The findings challenge universal approaches to physical activity promotion in older age by illustrating that a complex interplay of changing bodies and changing social relations over the lifecourse influence perceptions of and relationships with the aging body in sport and physical activity. We also raise questions about the value placed on being physically activity in the promotion of active and healthy aging. We provide a brief background to the Health and Sport Engagement (HASE) project. The coproduction and co-design strategies are introduced and we outline our methods to emphasize that they underpin the generation and status of the knowledge produced in this study. In the discussion of the findings we demonstrate that a complex interplay of sociocultural and political issues shaped the design, delivery, and participant experience in this project including issues of embodied aging, sociocultural determinants of health, stigma, life transitions, and the emotional aspects of engaging in sport in older age. The findings show that there is no single theoretical reason determining how older people make sense of and become and stay involved in physical activity. Rather there are a series of real issues which overlap and reflect existing literature on the lived experience of aging which are located in the field of aging embodiment. The insights we present can inform future work on community sport in later life by sensitizing researchers, policy makers, and practitioners to the complexities of the sporting experiences of older people.

## The Health and Sport Engagement Project: Background and Rationale

The current and heightened political ambitions in the UK to increase population levels of physical activity for health and well-being outcomes have become established in national sport strategy and funding models Department for Culture Media Sport DCMS., 2015; Sport England, 2016. Such approaches reflect post-London 2012 Olympic and Paralympic legacy strategies for increasing population level physical activity through sport (Cabinet Office, 2014), wider UK and global policy promoting physical activity for health (World Health Organization, 2010; Department of Health, D. H., 2011), as well as recognition by the UK Moving More, Living More cross-government group of the role that sport can play in helping people to become more active (Mansfield et al., 2015). The potential of sport to engage older people for health benefits is made explicit in such strategy. Sport England's current “Active Aging” fund, for example supports projects engaging with older people in developing sport opportunities to address health issues in later life including dementia, loneliness, and addiction.

These political, funding, and research agendas on sport and public health are not entirely new and they are not without critique (see Mansfield, 2018). Currently, critical debate prevails about the capacity of sport programmes to raise population levels of physical activity for health at any stage in the life course (Gard and Dionigi, 2016). The health benefits of sport for older people tend to be overemphasized (Dionigi, 2017) and sport participation for older people is dominated by a rather more exclusive demographic than the promotion of inclusivity accounts for because it is predominantly, white, wealthy and highly educated (Gard et al., 2017; Dionigi and Litchfield, 2018). Sport remains an activity associated with social exclusion of marginalized groups in society who experience complex barriers to engagement including poverty, challenges of time, (dis) ability, illness, lack of access to facilities and services, and family/cultural restrictions (Oliver et al., 2016), all of which may influence and be influenced by age and aging. The evidence-base on which to claim positive health benefits from sport is limited (Weed, 2017). Whilst policy goals and Government promotional campaigns suggest sport is a low cost, simple, and flexible solution to population health problems, the relationship between sport and health improvement is more complex (Waddington and Smith, 2013). In critiquing current evidence and policy agendas on sport for public health, and in considering how any potential health benefits of physical activity through sport are to be accomplished in an aging population, an alternative strategy for promoting and supporting the design and implementation of, access to, and engagement with community-based programmes is relevant. The Health and Sport Engagement Project (Mansfield et al., 2015) recognized that the potential health and well-being benefits of community sport can only be realized by addressing diversity, difference and inequalities in local populations and by employing collaborative partnerships approaches that involve local people in the co-design and implementation of projects.

Commissioned by Sport England (2013–2016) the HASE project was delivered with communities in the London (UK) Borough of Hounslow (LBH) and was a collaborative partnership between local authority commissioners and deliverers, researchers and local communities seeking to become more active. Drawing on community approaches (National Institute for Health Clinical Excellence NICE., 2009; World Health Organization, 2013; South, 2015) and the principles of coproduction, the HASE project included extensive formative research, participatory strategies and a theoretical and practical focus on understanding peoples' experiences, social norms, inequality, and their impact on engagement in community sport projects (O'Mara-Eves et al., 2013).

## Strategy and Methods for Coproduction in the Health and Sport Engagement Project

Our methods were mixed and included 18 months of formative involvement by the research team with local authority commissioners, deliverers, public health professionals, community groups, and workforce trainers to determine the overall focus of the project and the research questions. This approach based on ethnographic principles of observation and participation provided the basis for our coproduction strategies. Coproduction in community projects refers to the involvement of end-users in design and delivery decisions that can improve the quality of services (Realpe and Wallace, 2010). We adopted more creative, participatory practices to go beyond consultation to facilitate the sharing of experiences and ideas (Donetto et al., 2015). This necessarily involved the development of different relationships between partners in community projects and equalization of the balance of power between those providing a service to those who use it. This is not intended to oversimplify the complex dynamics of power, negotiation, and meaning in coproduction. There are challenges in taking a stance of reciprocity in partnership projects (see Mansfield, 2016). Maintaining transparent, honest, and mutual exchanges of information is challenging. However, we argue these must be central principles of co-design suffusing a range of decision-making including prioritization of research agendas, design principles, and activities, agreeing evaluation approaches and reporting findings.

Through this work, we gained insights into the pattern of aging in the LBH and community provision for this demographic. Joint Strategic Needs Assessment (JSNA) documents of the time (2012/13) identified a growing older population with the number of people aged over 85 years in Hounslow set to nearly double in the following 20 years, from 3,673 in 2012 to 6,075 in 2031 (an increase of 65% in a population of around 244, 926). The older White population were the dominant demographic living in 28 sheltered housing facilities for people over 50 years old and being provided with supported independent living opportunities through the work of resident wardens and scheme managers. The likelihood of increased demand for sheltered accommodation between 2014 and 2018 was identified and the need for a combined approach to ensuring high quality housing provision and living conditions that promoted health and well-being, independent living and positive community relationships was a core local strategy (London Borough of Hounslow, LBH., 2014) Recognizing the need for an expert workforce to deliver to the strategy, the “Communities Activities Coordinator” (CAC) was appointed to develop community physical activities for older people. Acting as the gatekeeper to the older population groups for our project we worked closely with the CAC to involve her in the project design, delivery, and evaluation.

During a 6 months collaborative development phase 10 community participatory focus groups were conducted. These involved 90 people aged 50 years and over, living across eight sheltered housing venues and including wider community groups. Not all these people took part in the project for its duration but figures from the HASE questionnaire used to measure project outcomes indicated that 81 participants were 50 years and older. In this group, most were female (74%), white (67%), had at least a GCSE qualification (64%), and 62% of these reported a low personal monthly income below £870. Of those participants 65 years and older (*n* = 68) again most were female (72%), white (69%), had at least a GCSE qualification (62%) and 65% reported a low personal monthly income below £870 (*n* = 65%). Focus groups used the principles of community participatory research in a collaborative approach based on reciprocal learning and taking action on findings (Macaulay et al., 1999). They drew on knowledge and expertise of potential participants about inactivity, sport engagement and community sport delivery. Participant knowledge was used to tailor community sport to the needs of older people. Lasting 1 h each and involving 6–12 older people at one time, the focus groups helped the research and community sport delivery team to get to know the older age inactive population of Hounslow and to unlock insights about inactivity and possibilities for becoming active through community sport. The discussion and activities emphasized four key issues: (1) memories of sport; (2) opinions/attitudes about sport; (3) practical experiences of physical activity; and (4) stimulus ideas and triggers for taking part in community sport.

To explore experiential aspects of physicality, participants were involved in four practical activities designed to give them commonly articulated potentially positive and negative experiences of taking part in sport and to examine participants views about their moving bodies. These included; a low-level physical activity experience to elicit a feeling of muscles moving (repeated standing up and down from a seated position); a deep breathing task to experience a potentially unusual breathing rate; a sport skill-based task (throwing and catching a ball or bean bag); and a competition activity (team-based target throwing game). Throughout the activities, participants discussed how they felt about taking part. In this way, the focus group work enabled inactive participants to connect their own past and present accounts of their inactive lives to a current experience of being physical and doing sport activities, and reconsider how this might impact on the potential for engaging in community sport.

Qualitative data were managed via NVivo 10 software, through the collation of tables using Word 2010 and/or via paper and pencil notebooks. Data was analyzed using the principles of thematic analysis. Qualitative data collection and thematic analysis of data analysis took place concurrently to ensure outcome, process and economic evaluations were effectively complementary and to enable the production of interim reporting to inform policy and practice (Smith and Sparkes, 2013). Analysis of qualitative data involved repeated reading, by two researchers (LM and TK), of focus group and interview transcripts, and observation notes to determine the details of the data and to enable researchers to identify key themes and patterns in it (Miles and Huberman, 1994; Attride-Stirling, 2001). Themes were identified by theoretical approaches focused on our analytical interest in aging embodiment and by inductive (data-driven) approaches drawing directly from the data produced. Coding frameworks were devised by two researchers (LM and TK). Discrepancies were resolved by discussion and the codes and themes verified by all researchers (LM, TK, NA, JF-R) to reflect the theoretical focus of the project and the research questions as well as salient issues evident in the data to support the process of identifying, refining, and interpreting key themes. Three overlapping themes were identified and inform a critical understanding of the constraining and enabling features of community sport for older people on low incomes and include a focus on: (1) lived experience, aging bodies and the changing dynamics of involvement in sport and exercise in the life course, (2) embodying aging—moving beyond practical barriers for understanding aging, lived experience and being physically active, and (3) corporeal pleasures of older sporting bodies. We use selected anonymized quotes in this paper to inform our understanding of the complex experiences of older people on low incomes who take part in community sport. In doing so, we critically discuss how their experiences reflect on the complex dynamics of aging, sport, and physical activity. We critically consider the implications of the findings for evidence-led policy and practice about older people and physical activity.

### Lived Experience, Aging Bodies, and the Changing Dynamics of Involvement in Sport and Exercise in the Life Course

Our discussions with and observations of older people in the project revealed the embodied nature of aging; a complex interrelationship between socio-cultural, political, and personal norms serving to construct and reconstruct how people make sense of, manage and present their bodies and themselves, and how they are perceived by others (Shilling, 2012). Aging is embodied and as the physical body changes over time so too do the habits, capacities, and meanings associated with them, and the identities forged through them (Tulle, 2007; Shilling, 2008). Such changes are at times connected to conscious action involving high degrees of autonomy but at the same time corporeal experience is shaped by factors beyond peoples' control (Lupton, 1995). Many of our older participants had previously led more active lifestyles than their current ones. Discussion about transition periods in life and the contexts in which they occur elicited diverse reflections and memories of how previous engagement and disengagement with physical activity and sport could occur. Such conversations highlighted that simplistic notions arguing past engagement in sport will lead to current or future participation habits are erroneous. Rather, relationships and the structure and organization of them, reflect the interplay between bodily change and social action and influence the nature of older peoples' physically active lives. Such relationships are also central to a sense of identity and the meaning attached to identifying as a physically active person in older age. Maggie recalled enjoying swimming in her youth before the changing demands of work and family responsibilities shaped her own activity in different ways:

*I loved swimming. I loved being able to do it. The water supports you… at school I did it … that pool in Chiswick we just dived in and I don't think that is allowed now…but then I was active [through] working and raising 3 children …I did take my kids swimming …then when they didn't need to go I didn't do it and then didn't do anything (60* + *F, sheltered accommodation)*

And Rod explained the way respiratory restrictions of living with Chronic Obstructive Pulmonary Disease (COPD) had halted his ability to take part in some sports in older age although not curbed his enthusiasm and internalized passion for activity saying “*I was a really good footballer–semipro–but too many fags and I've got COPD now ….couldn't do that now …I've done nothing for years ….no one our age here does that sort of thing…but I love all this and I'm doing it all–the swimming, skittles, all of it–probably too much (60*+ *M, White, Sheltered Accommodation)*.

For Maggie, a more self-defined autonomous relationship with her younger body reveals the corporeal pleasures of being capable of swimming and the positive feelings of freedom in the unrestricted ability to dive into the pool and take part with her friends. The example also illustrates though the heightened influence of working arrangements and family commitments in changing the relationship Maggie had with the swimming endeavor. For a time, she took her children swimming, teaching them the skills but withdrawing her own physical participation at the same time. This withdrawal from sport over time was reinforced as she grew older when the need to take her children faded from her life and the habit of swimming was no longer a part of her life. For Rod, the experience of aging was characterized by the severe impact of respiratory disease, restricting breathing, and the bodily capacity for physical exertion. Such a health crisis resulted for him in withdrawal from an active lifestyle; a process involving an acute redefinition and reestablishment of identity in old age (Phoenix et al., 2005; Dionigi, 2006; Shilling, 2008). Physical activity is commonly excluded from such corporeal changes because there is limited creative thinking about adapting the organizations and structure of opportunities to be active in older age and for those living with illness and disability (Williams and Smith, 2018).

Such complex ebbs and flows in the connection between life, corporeal change, and social action were further expressed by Joan. She discussed her experience in the Armed Forces as a more active time in her life and reflected on her doubts about her ability to be active in older age but also her desire to be active when she said “*I was in the army but age does catch up on you and you wonder [now] if you can do it…be active … but…it's good to try something [physical] out or try something again…if there is a chance to….we don't get much chance”(70*+ *F, sheltered accommodation)*. Here, the organizational stimulus for physical activity had been shaped by the practices of being in the Armed Forces, well-known for a focus on youth and peak physical performance. Yet, the lived experience of aging for Joan reveals such habits are not fixed and stable. An older life of inactivity had bought with it doubts about her ability to be active. In her comments about lack of “*chance”* to take part Joan also illustrates some of the challenges that older people face in being able to intentionally take control of their physically activity lives.

Eschewing a linear life transitions approach which suggests the same principles of transition apply throughout peoples' life and are definable by major life changes (e.g., school changes, marriage, parent-hood, and becoming a grandparent for instance), Phoenix and Tulle (2017) identify a complex dynamic of involvement in sport and exercise over the life course which the participants in our study revealed. Cowan and Hetherington's (2013) suggestion that transitions involve long-term processes characterizing a reorganization of feelings, habits and behaviors; shifts in how individuals understand and feel about themselves and the world, clarifies how our participants narrated their physically active and inactive lives. Maggie, Joan, and Rod's comments do indicate that major life changes impact on sporting engagement. Rather than reflecting a simple chronological explanation, however, their stories indicate that emotions, actions, and habits are influential in engaging in sport and physical activity (Williams and Bendelow, 1998; Shilling, 2008). They also signal the potential for re-engaging in such activity at any time of life which we discuss in more detail later in the paper. Most often the older people in our project did not know precisely how and why they had become more inactive. As Ralph explained “So *I was an active man, I think we all were….we need more support now…you think of sport different when you are young …like I said…it would take someone to really lead it …some of the equipment and the space is here…why did I stop? Lots of reasons really” (M, 65* +*, white, sheltered accommodation)*. Reasons were complex and shaped by the dynamic of life events, alterations in sporting provision, shifting ideologies of sport participation and changing bodies across the lifecourse into old age which served to redefine the pattern of their engagement.

Phoenix and Bell (2018) identify the ways that multiple bodily rhythms contribute to a sense of how activity and inactivity are defined, desires to be active, and opportunities and confidence to engage in physical activity in older age. These factors have very little to do with individual motivation as current established behavior change theories and policy agendas suggest. Slower rhythms of movement represented the reality of older peoples' engagement in sport and physical activity in our study. As Urmila explained her embodied experience of yoga was defined by a gentle pace of movement which she explained in health and well-being terms “*with yoga.you take your time …you don't count the time …like [you would] in most sports things ….[in yoga] you take it …you do it every day.you do your whole routine.however long it takes…and it keeps me strong in my body and well in my mind (F, 70*+*, Indian, sheltered accommodation)*. There is a challenge here to dominant ideas about the expected and established rhythms of physical activity which, in UK and global policies, are based on achieving defined amounts of daily exercise (Phoenix and Bell, 2018). Rather, our participants explained the complex ways that physicality, and the structures and processes of everyday life intersect. Their experiences show that defining physical activity simply by single bouts of exercise at predetermined levels of intensity is likely to prioritize the public health outcomes of physical activity for older people at the expense of wider impacts including on well-being, social connection, and friendship. Following Evans and Sleap (2015) our work reflects the idea that old age is one of many life stages all of which are interdependent with one another. It is not just the passage of time that marks out the aging process. Social, spatial, and sensory experiences of everyday life and of sport and physical activity through life reflect enabling and constraining features in the long-term dynamic of involvement in sport and exercise. This kind of complexity is excluded from the politics, policies and practices of the active aging agenda as we discuss in the next section.

### Embodying Aging—Moving Beyond Practical Barriers for Understanding Aging, Lived Experience, and Being Physically Active

Our findings did reveal practical barriers including time and cost constraints, most often cited in policy and physical activity guidelines, which discourage low income older people from being active (see for example Lim and Taylor, 2005; Sun et al., 2013). Our observation notes include comments on the potential unnecessary costs of sporting activities viewed as not essential to everyday living such as “*well I do have to think about my money* …*it's food and my washing items I need really…not sports ones” (F, 65*+*, white sheltered accommodation)*, and Patricia noted “*I don't expect things for free …but they need to be good value… you know not a waste of my money.if it benefits me I'd be willing to pay (F, 65*+*, white, sheltered accommodation)*. In addition, some of our participants revealed their daily lives to be full of domestic, leisure, work, and family activity presenting time itself as a barrier to engaging in community sport. For example, Mary explained “*…so it couldn't ever be when the bingo is…I run two lots of bingo and a coffee morning and we all do that” (F, white, 65*+ *sheltered accommodation)*. Others, like Doreen also identified existing community activities which would prevent people from taking part in community sport if it was delivered at the same time “*there is a singing group ….Tuesday – it could not be then…it's really popular … there's no point clashing ” (F, 65*+*, white, sheltered accommodation)*. Such individual reasons often connected to motivational approaches in policy and practice surrounding active aging have become established as key issues for resolving the acclaimed inactivity problem in older age populations. However, attention toward such practical barriers obscures the reality and complexity of aging, erasing the lived experience and providing uncritical and oversimplified policy and practice strategies in the promotion of sport and physical activity to older people. Multiple practical limitations to physical activity were only part of the picture of inactivity to emerge in our project. None of our participants suggested that adjustments to the scheduling and costs of activities alone would be enough to encourage them or support them to become active. Instead, most also expressed personal narratives of the way complex lived experiences shaped their lives and indeed their biographies of physical activity. For some, the narratives were of personal fulfillment and good health. Counter to established notions conflating old age with withdrawal from social interaction and activity and a growing dependence on others and the State, some of our participants led independent lives full of meaningful activity. Rani explained “*so yes I'd like to get involved but my days are so active with shopping and cleaning and visiting my family and there are lots of community projects I go to” (F, 65*+ *Indian, sheltered accommodation)* and Sara proudly stated “*I'm a racehorse. I never stop. I look after my grandsons. I go to the shops every day, I go see my daughters. I'm never home” (F, 65*+*, white, sheltered accommodation)*. To some extent such older people reinforce the morality of a “busy ethic” (Ekerdt, 1986, p. 239) similarly articulated in (Katz, 2000) account of the successful older citizen purporting the virtues of industriousness and self-reliance. Yet they also represent more self-defined articulations about what being active means to them and the value placed on everyday social and physical activity with friends and family.

Such examples illustrate that everyday physical activity reflects the interaction of peoples' experience of the aging body with their social and environmental milieu. Rani explained her busy life as making her feel “fulfilled,” “alive,” and “productive” as her years advanced. Similarly, Sara noted “you have to keep active like this—you feel more alive when you move your body and connected to people.” These views signal that in older age there remains an orientation to physical activity although it may not be defined by prescriptive guidelines and recommendations on its duration, frequency and intensity. Furthermore, such physicality, framed as it is by social connectedness illustrates the connections between community, meaning and individual identity in the embodiment of a sense of older-age. Habitual physicality and its direct association with physical activity provides an example in this study of Shilling's (2008, p. 12) ideas about embodying social action through routinized modes of behavior. The older participants in our study negotiated and renegotiated routine everyday physical activity as they managed the processes of aging, adapting to changes in their aging bodies and in terms of the relationship of their bodies, their environments and other people. As Mary emphasized “* well look – who can do what you did in your 20s, but it doesn't matter – I can get the tea and organize the Bingo and go to the shops every day and all of that and I am active (F, 65*+ *white female sheltered accommodation*). Such examples illustrate the enabling features in the capacity of some older people to successfully adapt to the biological and social corporeal markers of change that characterize aging. For those people, “habitual continuity” (Shilling, 2008, p. 12) in physical activity appears to expand the experience of positive well-being, extend the scope of relationships and create more potential for people to take control and make a difference to the world in which they live as they age. Here, as Urmila (F, 65+ Indian, sheltered accommodation) explained it, community sport and physical activity provide a site for resistance to both the perceived biological decline but also to the stereotype of frailty in older age: “*I do yoga for 2 and half hours every day now. In my room. It helps every aspect – walking and breathing and yes strength. I never use lift to 2*^*nd*^ *floor. I started yoga only 7 years ago. I am strong and healthy and calm…I share that with others”*.

Yet, aging can be characterized by times of physical and social crisis. For others in our study, we heard narratives of complex corporeal challenges and intersecting health and social inequalities that impeded the lived experience of aging and physical activity. The social gradient of health is a well-established characteristic of human life explaining that better health is associated with higher socio-economic status (Marmot, 2005). Despite the prevailing policy focus on biological and psychological determinants of ill health, there has been recognition that there are social determinants of health including housing quality, job quality, access to health care, welfare provision, unemployment, the quality, price, and availability of food and living environment (Marmot et al., 2008; Bambra et al., 2009). The impact of community disadvantage discussed by some participants revealed the influence of low income and poor social networks in shaping their capabilities to become involved in community projects. Monica, *(F, 70s, White British)* a widowed participant with limited physical capacity explained

where I was living on the Estate…unfortunately I have a son who is an alcoholic….I couldn't live like that anymore or do anything there…they wanted to get me in here ….to support ….a better place ….to help me and I've been doing all these activities now

In Gordon's comments there is also an illustration of the inextricable connection between the lived experience of aging on a low income, living in sheltered accommodation and facing environmental changes beyond the control of individuals which impacts on physical activity opportunities and engagement

*I have a list of fifteen activities that the warden is trying to run. [People] want to keep them but council is canceling them. Something isn't going right… we need a scheme manager who is living here but they want to take that away. We need support…and money …for all things like….activities… (Gordon, M, 65*+*, white, sheltered accommodation)*

The complex, diverse and intersectional nature of circumstance and experience, marked as it is by the social determinants of health influenced our participants' views about sport, their levels of inactivity and engagement in physical activity in older age. Counter-narratives to the inevitability of decline in old age have been illustrated in this paper. Yet, many of our participants did experience aging as physical decline including the debilitating impact of joint pain, reduced mobility, and respiratory difficulty coupled with wider issues of the low-income social conditions in which they lived. As Alf summarized it

*I have a walking frame. I'm housebound. I walk around every half hour. Also, I come down here [to the common room]. our old age expenses are limited… for us commitments are difficult – doctors, shopping etc. The problem is getting [physical activity] into peoples' mind [so we know] when, where and being regular….can you show what we need here (M, 65*+ *white, sheltered accommodation)*

These examples illustrate that alongside habitual corporeal continuity, corporeal crisis can characterize the aging process (Shilling, 2008). By this view, there is a disconnect between the body and its social and physical surroundings which render physical activity habits impossible and destroy what was once perceived as the norm. As Ralph explained:

not much goes on in our accommodation though… some people don't come out their rooms …I don't know ….not much to come out for anymore …. there was activities once I think … look there is a snooker table there never used …darts used to happen I dunno I suppose it would take someone to get it going….other people have health problems like me…breathing you know…makes it really difficult now (Ralph M, 65, white sheltered accommodation)

In these situations, sport never represented a panacea for ill health. Rather, there were strong expressions that older people felt like outsiders in community sport. Their affective responses to sport, largely featuring feelings of shame and embarrassment at believing they would to be incapable of taking part, indicated that physical health problems framed a sense of stigma about both the aging process and physical activity participation. Frailty, dependence and poor physical and mental health status were identified in our study as categories of stigma in old age. These markers of a spoiled identity (Goffman, 2009) appeared fundamental to older peoples' marginalization from sport and other organized physical activity. As Barbara explained:

*I thought about going swimming…then I remembered you'd need a costume with swimming …. I worried about looking out of place, arthritis, confidence, body-conscious and age …. and my husband died.I was depressed …not in the right mind to do anything like this (sport)….I could be in a better place now (F, 60*+*, white, sheltered accommodation)*

The stigma of aging in sporting contexts was more complex than individualized accounts suggest. Long-term processes of stigmatization associated with poor experiences of sport and perceptions of incapability meant that many older people who were currently inactive held deep-rooted negative perceptions about participating in sport. As Margaret stated “*My dreadful memory is of school sports day where I always came last or fell over (F, 60*+*, white, sheltered accommodation)*. They frequently expressed concerns about their personal capability and competence and compared themselves unfavorably to those who were or are “sporty” (i.e., good at sport and playing regularly and successfully). For these participants, processes of exclusion from sporting activity were remembered from their youth and were framed by networks of gossip surrounding who was capable and who was not. Such experiences of stigmatization produced a repertoire of negative emotions including fear, frustration, and anger but shame and embarrassment appeared to be the affective drivers (Elias and Scotson, 1994) of non-participation. In Maureen's words; “*I had swimming lessons when I got older …but it was the same …. I was afraid …I don't like it [because] I can't swim …I'm scared of others jumping in or passing me … it makes me feel bad (F, 65*+*, white, sheltered accommodation)*. Adults in older age whose identity is characterized by frailty and declining physical capacity find the thought and act of becoming physically active extremely challenging. Coupled with material deprivation, these social conditions of life present further difficulties in engaging in community sport. And yet, despite such deeply held and negative feelings, there remained a possibility of taking part for these participants. Re-establishing identities in this community sport project included the possibility of more pleasurable embodied experiences through physical activity in later life.

### Corporeal Pleasures of Older Sporting Bodies

Negative opinions about sport often resulted from taking part in traditional, regulated, competitive team games at school at a young age, usually as a compulsory activity. More receptive views were held by our older participants toward a wider programme of community sport when they were engaged with the participatory sporting activities used in the focus groups. These activities allowed them to experience the physicality of their older bodies, to embody a more positive experience of physical activity even for a short time. It signaled a moment for more optimistic feelings about their physical prowess and a sense of possibility in the community sport endeavor. Through this method, many expressed interest in becoming active if provided with a diverse range of sport-related activities, delivered through creative mechanisms and a co-design strategy to reflect the realities of older peoples' lives and their self-defined sense of physicality. As Joan stated *I'd like to be more active …I mean I like the sound of that skittles and bowling you were talking about ….and I like to walk …all that is something I could really get involved in.so long as it was something I could do and choose (F, 60*+*, White, sheltered accommodation)*. Also evident from our conversations with the older people who engaged in the participatory sporting activities was that more immediate, in the moment experiences of sport could invoke a corporeal reflexivity in which unexpected positive experiences of physicality in old age could be realized. The participatory elements of our focus groups provided an opportunity for participants to (re)engage in sport related activities in a way that enabled them to reflect on the possibilities of physical activity that sport can bring. This was not always easy or obvious, and a range of competing emotions were evoked in the experience, yet the perceptible idea of a physical potential in older age was evident. Harry explained the experience of a skill-based activity saying: “*I was worried when you asked us to throw and catch the ball …I didn't think I would be able to do it ….I did it …that could be quite a fun challenge” (60*+*, M, White, Sheltered Accommodation)*. In terms of an activity that simulated a deeper breathing experience that many people, young and old suggest they do not like about physical exertion, Myra noted “*I like the breathing exercises … I didn't realise you could do good exercise sitting down…[I feel] I am being active then” (70*+ *F, White, Sheltered Accommodation)* and similarly Ellen *explained “I don't like getting out of breath but that breathing is good … it makes me feel alive …I can feel all the air and oxygen going round my body …I am active breathing (F, 70*+*, White, Sheltered Accommodation)*.

Participation in physical activity was characterized by a broad emotional expression of pleasure in our older participants involving a swell of mixed feelings. Excitement, joy and happiness were commonly noted in relation to a sense of confidence and achievement. For example, Ann was involved in our swimming project and at her first session she exclaimed “*Wow I haven't been in a pool for 50 years … this is amazing …look at me …(nearly) swimming again” (F, 60*+*, white, sheltered accommodation)*. Rod also expressed his sense of joy at the swimming pool by splashing wildly and exclaiming “*wo-hoo …who would have thought I could do this again …I love it …so brilliant …I feel 15 again.”* By Shilling's (2008, p. 19) view such examples signify a more creative aspect to corporeal change in older life in which a redefined sense of self and environment serves to “repair or enhance one's embodied capacities for action.” This reflects other work that illustrates the potential for athletic competence through sport in later life and evidence of counter stories from sport to stereotypical ideas about decline in older age (Tulle, 2007, 2008; Phoenix and Smith, 2011). Our findings illustrate that pleasure is not simply an abstract emotion. There is a social dimension to pleasure which takes meaning through the relationships that people have in the context in which pleasure is felt. An ability to feel pleasurable emotion reflects the interconnection between the physical, the social and the psychological (Maguire, 2011). Emotions are socially constructed. The pleasurable character of the sporting experiences our older people expressed were inextricably connected to co-creation of a sporting experience that was underpinned by social interaction. The social interaction experienced through taking part in the sport activities they had co-designed with us reflected the realities of older peoples' lives in terms of the scheduling, delivery style, pace, timing, and type of activities, the use of appropriate space and place and personal support. There was a degree of routine in the organization of the activities. However, the elicitation of pleasure appeared to come from the opportunity to engage in activities that were identified by our participants as different, new or novel, and offering a break from their normal routines. Margaret noted “*I like it when there is a different activity each week … more exciting …bit of a challenge you know” (F, White, 65*+*, sheltered accommodation)* and Doreen explained

“*you see I think what brings us together is the different activities …it's just different —nothing like we'd normally do….the skittles, the indoor curling ….that instructor is fantastic….and we've tried table tennis…we would never do these things and the support for everyone means it doesn't matter if you aren't very good …it's just a great feeling to take part” (F, White, 65*+ *Sheltered Accommodation)*.

Like the participants in Humberstone and Cutler-Riddick's (2015) study of yoga, the older people in our project did not emphasize or take meaning from an ideology of sporting performance or prowess. Accepting the aging body but rejecting the idea of its assumed limits, our older participants took pleasure and an embodied a sense of meaning through community engagement in sporting activities. Such meaning making took place through a sense of accomplishment and joy felt in taking part, or watching others, trying new sports and being in the company of other people who they could relate to. Pleasure is not simply related to individual engagement in sport but is connected to the social relationships engendered in the sporting experience. The significance of the intimate connection between corporeal experience and social action came to the fore in Alf's explanation of another participant's experience:

“*see over there – that's Derek and he is deaf and I don't think I've seen him in here before because no one speaks to him. But that lad – the coach is signing to him …because he is deaf too…and I think it's the first time in ages someone has communicated with him and look at his face he is loving this” (M, 65*+*, white, sheltered accommodation)*

Our participants also illustrated that the sporting activities offered an opportunity for a more un-restrained, yet socially permitted flow of emotions that defines such activity as pleasurable (Maguire, 2011). In our observations of skittles, indoor curling, table tennis, and swimming, we commonly noted the laughter, loud chattering, opportunity for conversations, clapping, and cheering. A heightened feeling of pleasure was also experienced in some of the sessions when participants chose to play music as they participated including songs from the 40, 50, and 60s which they sang along to. There was a perceived potential for sport and physical activity to offer opportunities to develop positive relationships, enhance personal well-being, make friends, combat feelings of social isolation and loneliness, and experience a sense of community connectedness. Not only were respondents more inclined to take part with people who were like them, but they experienced a strong sense of pleasurable excitement if it was promoted as an opportunity for social connections (see for example, Heuser, 2005). The sporting activities offered a form of communal enjoyment through a playful type of physical activity. Pleasure was not only derived from taking part but also from watching and being with others. As Mrs Kaur noted one week when she arrived at the swimming pool “*I didn't feel well today … I am not swimming today.but I wanted to come out ….be with the group …I am pleased to be here.with people …I got up and out and moving” (F, 70*+*, Indian, Sheltered Accommodation)*. Others like Lesley emphasized the wider social role of community sport saying “*taking part is super….[but] make sure there's tea ….some time for talking …(F, 60*+*, White British, Sheltered Accommodation)*.

The older people in our project show that sporting activities can provide opportunities for pleasure through the ability to engage in playful activity which is distinctive and different from any usual routine. Such experiences elicit a sense of purpose and meaning, which participants viewed as health giving and connected to a positive sense of well-being irrespective of the intensity of physical activity involved. We emphasize here the potential greater significance of community sport as a place for identifying with a social group and experiencing pleasurable emotions in older age compared to the public health outcomes of disease prevention. As Eman (2012) has noted, sporting engagement in older age may allow people to make sense of the aging process and re-define a personal capability model of aging that challenges the idea of certain decline in old age.

## Conclusions and Implications

We recognize the tension in being involved in a physical activity project that includes making recommendations for decision makers likely to be part of an established system of promoting and prescribing physical activity for health in older age. However, our work demonstrates that experiences of physical activity in older age go beyond the pursuit of public health as the absence or risk reduction of disease. Furthermore, the coproduction strategies which framed the project provided a forum for effective communication of findings which could challenge decision making and practice to an extent. The project moved beyond telling older people to be active and providing opportunities for activity that were not designed with involvement from participants. Our approach was to explore the significance of locally specific public knowledge (Kay, 2016) from older people with limited income which directly addressed the complexity and inequalities of their everyday lives. Such lived experiences are most likely to impact on preferences for, engagement in and enjoyment of physical activity.

Our findings illustrate that aging and experiences of community sport and physical activity in later life are shaped by a complex interplay between changing bodies and the dynamics of relationships in differing social contexts. The lived experience of aging involves continuity and change in capacities and opportunities for physical activity and sport that are not fully recognized in the policy landscape of active aging. Moreover, the additional challenges and constraints affecting those on low income are notable only for their omission. Experiences of corporeal crisis through illness, disease, and disability that disproportionately affect lower income adults are annihilated in established biomedically informed policies for physical activity in older age. Such policies rather simply and universally emphasize certain frequencies, intensities, and types of physical activity in older age for disease prevention and symptom alleviation. The pleasures and creative potentials of different forms and types of engagement in sporting and other leisure activities including taking part, watching, socializing with others, and experiencing welcomed moments of solitude or calm are marginalized. The findings in this study suggest that policy and practice agendas for sport and physical activity in old age need to redefine what physical activity for health and well-being means in this population group, recognizing especially how constraints experienced in older age may be exacerbated among those with low income. A broader definition of physical activity is needed that recognizes the value of activities that will vary in the type and degree of physicality they involve yet still provide opportunities for positive individual and community experience for older people.

## Data Availability

The datasets are not publicly available because data from the project is being prepared for a range of publications. Requests to access the datasets should be directed to louise.mansfield@brunel.ac.uk.

## Ethics Statement

This study was carried out in accordance with the recommendations of the Brunel University London Research Ethics Committee with written consent from all subjects. All subjects gave written informed consent in accordance with the Declaration of Helsinki. The protocol was approved by the Brunel University Research Ethics Committee and is published.

## Author Contributions

LM, TK, NA, and JF-R contributed to the design of the HASE Project in collaboration with the project partners. All the authors contributed to the analysis and interpretation of study data and drafted, critically appraised and approved this manuscript.

### Conflict of Interest Statement

The HASE intervention was co-designed by Brunel University investigators, and London Borough of Hounslow local community participants, sport deliverers, and public health professionals. The project was funded by Sport England and Brunel University investigators evaluated the project. LM and TK are members of Sport England's Advisory Board on Evidence. The remaining authors declare that the research was conducted in the absence of any commercial or financial relationships that could be construed as a potential conflict of interest.
